# Findacure – the Fundamental Diseases Partnership

**DOI:** 10.1186/1750-1172-9-S1-O23

**Published:** 2014-11-11

**Authors:** Anthony K Hall, Nicolas T Sireau

**Affiliations:** 1 Findacure: the Fundamental Diseases Partnership, AdviceSpace, 66 Devonshire Road, Cambridge CB1 2BL, UK

## 

Findacure is building a movement to promote the search and development of treatments and cures for fundamental diseases, on behalf of patients and those who care for them.

Fundamental diseases are extreme and rare genetic disorders that offer a unique opportunity to better understand other diseases, including many common conditions. For instance, study of the LDL-receptor in familial hypercholesterolemia led to the development of statins for the prevention of heart disease. Research into the articular damage in alkaptonuria, a rare genetic disorder, has led to be a better understanding of osteoarthritis

Findacure empowers patient groups to evolve into effective advocates for change. Findacure also campaigns for a receptive research environment and facilitates patient groups to drive the development of treatments.

If a patient group exists, it’s often run privately and part-time by patients or patients’ family members. They generally have little scientific background, few contacts in academia, no knowledge of drug development, and limited experience of fundraising. That’s why Findacure is organising a series of workshops with expert speakers covering key issues for new patient groups, such as how to participate in clinical trials, how to interact with industry and academia, and how to manage a small patient group effectively.

On the drug development side, a good example of Findacure’s partnership model is the DevelopAKUre programme.

The main objective of DevelopAKUre is to study the efficacy and safety of an orphan designated drug, nitisinone, in order to obtain its marketing authorisation for the treatment of patients with alkaptonuria, for which there is no licensed treatment.

The DevelopAKUre consortium brings together a pharma company, a biotech, academia, clinicians and two patient groups in a new model of collaboration. It applied for funding from the European Commission in order to implement phase 2 and 3 clinical trials. To do so, it had to overcome a number of regulatory, scientific and logistical hurdles – many of which are faced by other patient groups trying to develop treatments.

Learnings from Findacure’s experience and its wider network will be published in a forthcoming handbook for rare disease patient groups early next year.

